# Impact of Age on Outcomes of Total Pancreatectomy: A Narrative Review

**DOI:** 10.7759/cureus.92708

**Published:** 2025-09-19

**Authors:** Rohan Maydeo, Harsh Patel, Bennet Duraisamy, Manoharan Govindhan

**Affiliations:** 1 Department of Surgical Gastroenterology, Dhanalakshmi Srinivasan Medical College, Siruvachur, IND; 2 Department of Gastroenterology, Dhanalakshmi Srinivasan Medical College, Siruvachur, IND; 3 Department of Surgical Gastroenterology, Dhanalakshmi Srinivasan Medical College Hospital, Siruvachur, IND

**Keywords:** age-related outcomes, pancreatic surgery, postoperative outcomes, surgical morbidity, total pancreatectomy

## Abstract

Total pancreatectomy (TP) is a complex and rarely performed procedure. With advances in surgical techniques, perioperative care, and islet auto transplantation, TP has seen renewed clinical interest. This narrative review explores the current evidence on outcomes following TP, with a focus on age-related differences in morbidity, mortality, metabolic challenges, quality of life, and survival. Relevant literature from peer-reviewed journals was reviewed to compare perioperative and long-term outcomes in younger versus older patients undergoing TP, including the role of total pancreatectomy with islet auto transplantation (TPIAT). Although TP is associated with significant metabolic and nutritional challenges, advances in perioperative management have improved outcomes. Older patients often experience longer hospital stays and reduced long-term survival compared to younger cohorts, though age alone is not a contraindication. Islet yields in children tend to be higher, while readmission rates and postoperative recovery patterns vary between age groups. Age influences several outcome parameters following TP but should not be considered an absolute barrier to surgery. Careful patient selection, individualized metabolic support, and multidisciplinary management are critical for optimizing outcomes across all age groups.

## Introduction and background

Total pancreatectomy (TP) is a complex surgical procedure involving the complete removal of the pancreas, often accompanied by resection of adjacent organs such as the duodenum, gallbladder, spleen, and part of the stomach or bile duct [1]. The initial documentation of TP as a surgical approach for pancreatic cancer was made by Rockey in 1943 [2]. Originally developed for the management of multifocal or diffuse pancreatic disease, TP is now selectively used in cases of extensive intraductal papillary mucinous neoplasia (IPMN), multifocal pancreatic neuroendocrine tumors, unmanageable chronic pancreatitis, and certain locally advanced or borderline resectable malignancies [3].

Historically, its application was limited due to significant perioperative morbidity and challenging long-term metabolic consequences, particularly brittle diabetes and exocrine insufficiency [4]. However, in recent years, refinements in the surgical technique, improvements in critical care, the development of advanced insulin regimens, and better pancreatic enzyme supplementation have led to a reassessment of the role of TP in pancreatic surgery. Functional and metabolic outcomes have gradually improved, and with careful preoperative planning, patients are able to maintain a reasonable quality of life postoperatively [5,6]. Although TP remains uncommon, accounting for less than 5% of major pancreatic resections in high-volume centers, its role has expanded in recent years [7].

Furthermore, age once considered a limiting factor has not consistently been shown to significantly impact long-term outcomes. Several studies now support the view that elderly patients can benefit from TP when rigorous selection criteria are applied, and comorbidities are appropriately managed [7]. Age is increasingly recognized as an important determinant of outcomes; older patients often present with more comorbidities and reduced physiological reserve, whereas younger patients face the challenge of long-term metabolic and nutritional consequences.

This narrative review synthesizes current evidence on TP, focusing on four main outcome domains, perioperative risks, survival, quality of life, and endocrine/exocrine function, while also discussing total pancreatectomy with islet autotransplantation (TPIAT) as a distinct subgroup.

## Review

Patient selection: indications and contraindications 

Indications for Total Pancreatectomy

TP may be considered in a variety of clinical scenarios, ranging from benign but debilitating conditions like chronic pancreatitis to complex or extensive malignancies. Indications for TP can be broadly categorized into three groups: established chronic or hereditary disease, recurrent functional impairment, and the "Four T’s" framework proposed by Janot et al. [8].

Chronic Pancreatitis and Functional Impairment

Patients with long-standing chronic pancreatitis may be eligible for TP if they experience persistent abdominal pain alongside one or more of the following criteria: radiological evidence of pancreatic calcifications, moderate to severe ductal abnormalities according to the Cambridge classification, histological confirmation of chronic pancreatitis, or recurrent episodes of acute pancreatitis linked to the chronic disease process.

Similarly, individuals suffering from two or more radiologically confirmed episodes of acute pancreatitis without an identifiable, reversible cause may be considered for TP if these episodes significantly impair quality of life or daily function. Additionally, patients diagnosed with hereditary or genetic pancreatitis may qualify for surgery, particularly when clinical features overlap with chronic or recurrent acute pancreatitis [9].

The "four T’s framework" (tumor, trouble, technical difficulty, therapy-refractory pain): Janot et al. [8] categorized the indications for TP into four domains. Tumor-related indications include multifocal or locally advanced pancreatic cancer, diffuse involvement by IPMN, recurrent pancreatic adenocarcinoma, and extensive neuroendocrine tumors, all of which render partial resections ineffective or technically unfeasible. “Trouble” cases refer to urgent or salvage situations arising in critical care, such as pancreatic stump necrosis, persistent anastomotic leaks, or hemorrhage due to vessel erosion, where conservative management fails; in such instances, elevated C-reactive protein levels above 100 mg/L can serve as early indicators of vascular complications. Technical difficulty arises when intraoperative findings such as a soft and friable pancreatic remnant make reconstruction unsafe, and in such cases, an elective TP may be performed to avoid higher-risk emergency surgery. Therapy-refractory pain applies to patients with chronic pancreatitis who continue to suffer from severe abdominal pain despite optimal medical and surgical therapy; in these individuals, TP may be the only option, with studies reporting pain relief in 30-60% of patients.

Additional Modern Indications

Advances in diagnostics and perioperative care have expanded the indications for TP. These include cancers involving the head, body, and tail of the pancreas when clear margins cannot be achieved through partial resection; inconclusive pathology or ductal abnormalities noted intraoperatively during frozen section analysis; recurrent malignancy in the residual pancreas following previous surgery; combined resections such as pancreatectomy with celiac trunk resection in selected advanced cancers; salvage procedures following severe postoperative complications such as anastomotic leaks leading to sepsis; multifocal or dysplastic IPMN with widespread gland involvement; isolated pancreatic metastases from cancers such as renal cell carcinoma or melanoma in the absence of systemic spread; multiple pancreatic neuroendocrine tumors, particularly in patients with multiple endocrine neoplasia syndromes; and high-risk hereditary cancer syndromes, where prophylactic TP may reduce the risk of malignancy (Table 1).

**Table 1:** Indications for Total Pancreatectomy

Category	Specific Indications	Age-related Considerations
Chronic or Hereditary Disease	Long-standing chronic pancreatitis with persistent pain and radiological/histological evidence. Hereditary/genetic pancreatitis with recurrent or overlapping chronic pancreatitis features	Younger patients with hereditary pancreatitis may be considered earlier; the elderly require strict comorbidity assessment
Recurrent Functional Impairment	Multiple radiologically confirmed acute pancreatitis episodes without a reversible cause. Severe impairment of daily function/quality of life	More common in younger/middle-aged patients; the elderly often have higher perioperative risks
Four T’s Framework (Janot et al.)	Tumor: Multifocal or diffuse IPMN, neuroendocrine tumors, recurrent adenocarcinoma, locally advanced cancers. Trouble: Salvage for pancreatic stump necrosis, uncontrolled anastomotic leak, hemorrhage. Technical Difficulty: Unsafe reconstruction due to friable pancreatic remnant. Therapy-refractory Pain: Severe pain in chronic pancreatitis unresponsive to maximal medical/surgical therapy	Applies across all ages; careful selection crucial in elderly, where comorbidities and frailty may affect outcomes

Contraindications for Total Pancreatectomy

Surgical removal of the pancreas is not recommended in situations associated with poor outcomes. Active substance abuse (ongoing severe alcohol dependence) and uncontrolled comorbidities are commonly considered relative contraindications to major pancreatic surgery. Although tobacco smoking is associated with increased perioperative pulmonary and anastomotic risks and worse oncologic outcomes, it is not generally regarded as an absolute contraindication to pancreatectomy [10].

Surgical considerations in elderly patients

Preoperative Risk Assessment in Pancreatectomy

Evaluating a patient’s preoperative risk profile is crucial for anticipating outcomes following TP. A large-scale analysis [11] using data from the American College of Surgeons National Surgical Quality Improvement Program (ACS NSQIP) from 2014 to 2017 examined the utility of three predictive models: the Universal Risk Calculator (URC), the Model for End-Stage Liver Disease (MELD), and the Modified Frailty Index-5 Factor (mFI-5). Among 22,123 pancreatectomy cases, the 30-day mortality rate was 1.4%, with complications occurring in 27.2% of patients. The URC demonstrated the highest predictive accuracy for both 30-day mortality and overall complications, with an AUC of 0.70 for mortality and 0.59 for any complication, outperforming both MELD and mFI-5. However, despite its relative superiority, all three models showed only modest discriminative ability, suggesting that while they are helpful, they should be used in conjunction with clinical judgment and patient-specific evaluation.

Age as a Determinant of Surgical Outcomes

The influence of age on postoperative outcomes following TP remains a subject of ongoing debate. Several studies have reported a higher incidence of postoperative morbidity among older patients compared with their younger counterparts, suggesting that advancing age may be associated with increased susceptibility to complications, possibly due to a greater burden of comorbidities and reduced physiological reserve [12-15]. In contrast, a retrospective analysis involving 428 patients found no significant differences between older and younger groups with respect to gender distribution, disease aetiology, or type of resection performed, and reported comparable rates of morbidity, mortality, and median hospital stay between the two cohorts [16]. Overall, while some studies highlight age as a potential risk factor, others suggest that chronological age alone may not be a definitive predictor of surgical risk or outcomes. These discrepancies emphasize the importance of considering functional status, comorbidities, and frailty, rather than age in isolation, when evaluating candidates for TP.

Perioperative Outcomes

Morbidity and mortality in different age groups: Reports on morbidity and mortality following TP have varied considerably. Morbidity has ranged from 31% in the study by Müller et al. [6] to as high as 87% in the report by Nikfarjam et al. [17], while mortality has been reported as low as 0% by Satoi et al. [18] and as high as 9% by Nathan et al. [5]. Reddy et al. [19] documented a morbidity of 69% and a mortality of 8%, whereas Bhayani et al. [20] reported rates of 38% and 6.1%, respectively. In a series of 428 pancreatic resections, Oliveira-Cunha et al. [16] observed a perioperative mortality of 2.8%, with no significant difference in outcomes between patients above and below 70 years of age. Similarly, Adham et al. [21] reported that age ≥70 years was significantly associated with higher postoperative mortality following pancreatectomy, with mortality rates of 12.9% in elderly patients compared to 3.9% in those under 70 (p = 0.003). While Müller et al. [16] and Nikfarjam et al. [17] reported widely varying morbidity rates (31% vs. 87%), these discrepancies may reflect differences in study design. Müller’s data were derived from a high-volume, single-center series with standardized perioperative protocols, whereas Nikfarjam’s cohort was more heterogeneous, spanning multiple centers and including patients with varied baseline comorbidities. Such methodological differences limit direct comparison. Hemorrhagic complications were the leading cause of death in the older cohort, contributing to one-third of fatal outcomes, followed by cardiopulmonary events. Univariate analysis identified age, intraoperative blood transfusion, blood loss, and type of procedure as risk factors for mortality, with age ≥70 remaining an independent predictor in multivariate models (p = 0.001). Interestingly, although older patients experienced fewer severe complications compared with younger individuals (12% vs. 28%; p = 0.04), their overall survival outcomes were worse.

ICU Stay, Length of Hospitalization, and Complication Profile

The study by Cerullo et al. [22] demonstrated that routine ICU admission following major pancreatic resection did not significantly affect outcomes such as failure to rescue or prolonged hospitalization. Patient-specific factors such as age and sex were not significantly associated with ICU admission or worse outcomes. Failure-to-rescue rates were comparable between ICU patients (3.7%) and those managed on the ward (1.7%, p = 0.098). Interestingly, however, the median hospital stay was longer for patients admitted to the ICU (10 days, IQR 7-15) compared with those managed on the general floor (8 days, IQR 7-12). Most studies involving patients with a mean age above 80 years report hospital stays ranging from 11 to 25 days [12,23,24,25], though few have directly compared outcomes between younger and older adults in this context. The most commonly reported complications include delayed gastric emptying (10.9%), and cardiopulmonary issues (9.3%), with a reoperation rate of 7.5% [7]. In another cohort, delayed gastric emptying was the leading complication, affecting 13.7% of patients, followed by pulmonary complications in 12.5% [26]. Older patients were significantly more likely to require hospital readmission due to malnutrition or dehydration, with an approximately eight-fold higher likelihood compared with younger individuals (p < 0.005) [27].

Long-Term Outcomes

Oliveira-Cunha et al. [16] reported that younger patients generally achieve the most favorable long-term survival following pancreatic resection, while older patients, particularly those over 80 years, experience markedly reduced survival, with the oldest group showing a significantly higher early mortality rate of 44% within the first year after surgery. Although age alone is not considered a contraindication for pancreatic surgery, these findings highlight the importance of careful patient selection in elderly individuals. Other studies provide conflicting evidence. A single-institution series from UCSF reported a postoperative mortality rate of 3%, with no statistically significant difference between elderly patients (≥75 years) and younger cohorts (<75 years), suggesting that chronological age may not independently predict outcomes. However, elderly patients demonstrated higher rates of complications, including pneumonia, acute renal injury, sepsis, pancreatic fistula, and altered mental status, and nearly half required enteral nutritional support at discharge compared with a smaller proportion of younger patients. Despite this intervention, malnutrition and dehydration remained leading causes of hospital readmission among elderly individuals [27]. Conflicting survival outcomes across age groups may be partially explained by study design. Oliveira-Cunha et al. conducted a retrospective single-institution analysis, which may underpower age-specific subgroup comparisons, while multicenter prospective analysis by Adham et al. [21] provided stronger evidence for age ≥70 as an independent predictor of mortality. These variations highlight the need for prospective, standardized, age-stratified trials.

Quality of Life and Functional Independence

Pulverenti et al. [26] evaluated quality of life in patients divided into three age groups after TP. The youngest group (≤55 years) reported significantly lower scores across all functional and mental health domains compared with the general population. The middle-aged group (56-70 years) also demonstrated reduced quality of life, although pain and general health perception were comparable with the general population, and only mental health showed a significant decline. Interestingly, in patients older than 70 years, only social functioning and mental health scores were lower, suggesting that quality of life in older adults is relatively preserved after surgery.

Nutritional Implications and Exocrine Insufficiency

Exocrine pancreatic insufficiency is common after major pancreatic surgery and contributes to malabsorption, weight loss, and deficiencies in fat-soluble vitamins unless managed proactively with enzyme supplementation. A large institutional series found that 36% of patients developed exocrine insufficiency and 20% developed endocrine insufficiency within approximately 14 months of surgery, underscoring the need for long-term follow-up [28]. These metabolic disturbances significantly affect nutritional status and quality of life, highlighting the importance of proactive monitoring and early intervention [29]. A recent systematic review involving 30 studies and 2,305 patients reported that the prevalence of exocrine insufficiency after pancreatic surgery exceeded 65% [30]. The faecal elastase-1 test was the most commonly used diagnostic tool. Despite its prevalence, pancreatic exocrine insufficiency is often underdiagnosed and inadequately managed, with enzyme replacement therapy frequently underutilized. For therapy to be effective, clinicians should consistently prescribe pancreatic enzymes and refer patients to a dietitian to ensure appropriate dosing and dietary management [31].

Age-Specific Metabolic and Compliance Challenges

Profound metabolic disruptions, including insulin-dependent diabetes and exocrine insufficiency, are common following TP [32]. These challenges are particularly complex in elderly patients, who often have reduced physiological reserve, cognitive decline, and multiple comorbidities. Glycemic control is more difficult to achieve in older adults due to impaired counter-regulatory mechanisms, increased risk of hypoglycemia, and reduced insulin sensitivity [33]. In addition, age-related decline in appetite, mobility, and nutritional absorption can exacerbate malnutrition after surgery. Although limited literature directly compares age-specific outcomes, available evidence and clinical experience suggest that older adults face unique metabolic and compliance-related challenges.

Total Pancreatectomy with Islet Autotransplantation

TPIAT is a distinct clinical procedure with different patient selection (usually chronic/hereditary pancreatitis in younger patients), goals (pain relief and preservation of insulin secretion), and outcomes (islet yield, insulin independence). Results from TPIAT series should not be extrapolated to TP performed for malignancy. The role of TPIAT appears to differ between younger and older patients. Bellin et al. [34] evaluated the procedure in very young children (ages 3-8) with severe chronic pancreatitis and genetic predisposition and reported high rates of pain relief, narcotic cessation, and excellent endocrine outcomes, with more than 80% achieving insulin independence and maintaining mean HbA1c levels below 6.5% on follow-up. In contrast, older children and adults achieved insulin independence in only about 41% of cases, suggesting that younger age is associated with superior islet graft success and glycemic control. The Prospective Observational Study of TPIAT (POST), led by Nathan, Yang, Eaton, and colleagues [35], compared surgical approaches and short-term outcomes between pediatric (n = 84) and adult (n = 195) recipients across multiple U.S. centers. Children demonstrated higher median islet equivalents per kilogram transplanted compared with adults, indicating superior islet preservation, despite COBE purification being less commonly used. While children experienced longer median hospital stays, their 30-day readmission rates were lower than those of adults, suggesting that age influences both the efficiency of islet engraftment and the trajectory of postoperative recovery.

Current Evidence and Gaps in the Literature

Several studies have explored the outcomes of TP with respect to age, yet age-specific analysis remains underrepresented. Existing literature provides some insight into postoperative survival, morbidity, and quality of life stratified by age groups. For instance, Oliveira‐Cunha et al. [16] analyzed over 428 pancreatic resections and found no statistically significant difference in perioperative mortality or morbidity between older and younger cohorts. However, long-term survival was notably poorer in patients over 80, as nearly 44% of them died within the first year postoperatively.

Similarly, Pulvirenti et al. [26] reported reduced physical and mental component scores in all age groups following TP, with the most pronounced impairment in younger patients (<55 years), potentially reflecting greater disruption of life roles. On the other hand, Cerullo et al. [22] demonstrated that age was not a significant predictor of extended ICU stay, length of hospitalization, or failure-to-rescue outcomes following major pancreatic surgery.

Despite these data points, the major limitation is the lack of robust, prospective, age-focused studies specifically evaluating TP susceptible to selection bias and under-reporting of complications. Most reports pool data from various pancreatic resections (e.g., PD, TP, DP) or do not stratify outcomes beyond basic age categories.

Discussion

TP, while historically considered a high-risk surgery, has become increasingly feasible due to advances in perioperative care and endocrine/exocrine management. However, balancing the potential benefits against postoperative burdens remains crucial especially in elderly individuals. This review highlights that age alone should not be considered a contraindication, as many older patients achieve acceptable short- and long-term outcomes. Instead, careful patient selection, multidisciplinary support, and individualized risk assessment are key.

Postoperative complications such as pancreatic fistula, delayed gastric emptying, and pulmonary events continue to pose significant morbidity risks [26]. Additionally, endocrine and exocrine insufficiencies particularly post-pancreatectomy diabetes and malabsorption demand vigilant, long-term follow-up and proactive management, including PERT and insulin therapy [36]. Nutritional deterioration is common but manageable with appropriate dietary intervention and patient education.

Age-stratified data demonstrate that while older patients may face higher early mortality, younger patients are more likely to report reduced quality of life postoperatively [16,26]. This discrepancy reflects differing expectations, lifestyle demands, and adaptability. Future research should focus on prospective multicenter studies examining age-related physiological resilience, adherence to therapy, and functional recovery.

## Conclusions

Elderly patients undergoing TP experience higher early postoperative mortality and increased rates of complications such as malnutrition and dehydration compared to younger patients. Despite this, morbidity and long-term survival outcomes were often comparable between age groups, indicating that advanced age alone should not be considered a contraindication. Younger patients reported greater impairment in quality of life, particularly in physical and mental health domains, following surgery. These findings emphasize the need for careful, individualized preoperative assessment focused on functional status and comorbidities to guide patient selection and optimize outcomes.
